# Effect of selective serotonin reuptake inhibitor treatment on the prognosis of patients with medication overuse headache

**DOI:** 10.1097/MD.0000000000010193

**Published:** 2018-03-23

**Authors:** Jinghuan Fang, Yang Zhang, Ning Chen, Jian Guo, Muke Zhou, Li He

**Affiliations:** Department of Neurology, West China Hospital, Sichuan University, Chengdu, P.R. China.

**Keywords:** medication overuse headache, preventive treatment, SSRI

## Abstract

Medication overuse headache is a disabling headache disorder. Withdraw treatment plus preventive medication may lead to a better outcome. However, the effect of selective serotonin reuptake inhibitor on the treatment of medication overuse headache is still unknown.

In this study, we followed up medication overuse headache patients diagnosed at the West China Hospital at an average follow-up duration of 1.5 years to analyze patients’ outcomes and relapse. We used logistic regression to assess the relationship between patient medication and effect of withdrawal treatment. We used COX (cox regression model) regression analysis to assess the relationship between withdrawal treatments and relapse rate in patients.

A total of 72 medication overuse headache patients were enrolled in this study, of which 14 (19.4%) failed to withdraw therapy, 58 got a good response to withdraw therapy. Among responders, there are 5 (8.6%) relapse patients. Selective serotonin reuptake inhibitor treatment can increase the effect of withdrawal therapy (odds ratio [OR] = 0.016, 95% confidence interval [CI]: 0.003, 0.091, *P* < .001) and it was an important predictor of patients’ outcome (hazard ratio [HR] = 0.255, 95%CI: 0.09–0.724).

Selective serotonin reuptake inhibitor can increase the therapeutic effect in medication overuse headache withdrawal therapy and can reduce the risk of relapse.

## 1 Introduction

Medication-overuse headache (MOH) is a common disabling headache disorder.^[1,2]^ The prevalence of MOH in the general population is about 1% to 2%.^[2]^ In the headache clinic, this figure increased to 55% to 70%.^[3,4]^ MOH gives patients great economic burden including loss of working time, drug costs, and the consumption of health resources.^[4–7]^

The best treatment for MOH is still controversial, however, most headache experts regard withdrawal of the overused medications as the best choice for patients.^[8,9]^ Although patients have received withdrawal therapy, some of them are still have headache without remission.^[10]^ Studies showed that early discontinuation with preventive medication may lead to a good outcome.^[11]^ Preventive treatment was chosen based on patients’ comorbid disorder.^[11]^ MOH patients have a greater risk of suffering from anxiety and depression.^[12]^ Moretti et al^[13]^ found that selective serotonin reuptake inhibitor (SSRI) (citalopram) may suggest an adjunctive therapy during withdraw therapy. However, the role and effect of SSRI in withdrawal therapy has not been confirmed. Moreover, the high relapse rate of MOH is another major treatment problem. According to literature, the relapse rate is 20% to 40% at the first year after overuse discontinuation,^[14–17]^ and 20% to 50% at the long-term duration.^[16]^ Predictors of MOH relapse are limited. Previous studies have reported that overused medication, duration of primary headache, type of primary headache disorder, and dependence levels may be the predictors of MOH outcome.^[16,18–20]^ To our knowledge, the effect of SSRI on treatment of MOH is still not to be confirmed.

The aim of our study is to evaluate the potential effect of SSRI treatment in MOH and the effect of SSRI on the prognosis of MOH.

## 2 Methods

### 2.1 Patient

We enrolled MOH patients between May 2014 and February 2017 at Department of Neurology West China Hospital and MOH diagnosis were according to International Classification of Headache Disorders, ICHD-3β. Study exclusion criteria were as follows: patients with other chronic pain require the use of analgesic drugs; patients with other chronic diseases (e.g., hypertension, diabetes, and other cardiovascular diseases.); patients with major depression disorder or anxiety disorder. The following data were extracted: demographic data (sex, age of headache onset, years of education, body mass index [BMI], coffee consumption, smoking, family history); the features of the chronic headache; type of overused medication as well as frequency and duration of drug intake; preventive treatment (type of medication, including SSRIs, tricyclic antidepressants, topiramate, and valproic acid). Headache intensity was measured by Visual Analogue Scale (VAS) 0 to 100.

We defined outcomes as followings. Responders (effective treatment): patients who had overuse free and headache days per month decreased 50% compared with the baseline. Nonresponder: patient who did not achieve overuse free at the first 2 months regardless of their headache pattern and relapser was defined as a patient who become responder in the first 2 months, but relapse to overuse at least 3 months.

The Institutional Ethics Committee of Sichuan University approved the study protocol.

### 2.2 Treatment and follow up

All patients were advised to withdraw overused medication by outpatient treatment. Patients were allowed to take non-steroidal anti-inflammatory drugs (naproxen or ibuprofen) as rescue medication. Preventive treatment was admission based on the patients’ characteristics. All patients were instructed to keep a headache diary. We followed up patients by telephone based on a standard questionnaire or by face-to-face interviewing in the outpatient department in West China Hospital. Follow up time points were set as: 1 month, 2 months, 6 months, 1 year, and 2 years. At visit 1 (1 month), visit 2 (2 months), and visit 3 (6 months) all patients were asked to come to our outpatient department in West China Hospital and interviewed and examined by neurological expert in headache. At visit 4 (1 year) and visit 5 (2 year), we followed up all patients mainly by telephone. At each follow up time point, we assessed patients’ headache features (frequency, intensity, medication, and headache days per month).

### 2.3 Statistical analysis

Continuous variables were compared using the Student *t* test. Categorical variables were compared using the chi-square test or Fisher exact test, as appropriate. Logistic regression analysis was used to test the association between SSRI use and effect of treatment. Cox regression analysis was used to test the association between SSRI use and relapse. All tests were 2-sided and *P* < .05 was considered significant. SPSS v24 (SPSS, Chicago, IL) was used for all analyses.

## 3 Results

We included a total of 103 MOH patients and followed up for a mean period of 1.5 years. Seventy-two patients have completed the study including 25 men (34.7%) and 47 (65.3%) women. Among them, 58 (80.5%) patients were responders and 14 (19.5%) were nonresponders. Among 58 responders, 5 (8.6%) patients relapsed.

In this study, more than half of patients (55, 76.4%) overuse more than one kind of medicine and we defined them as poly-overuse (Table 1). The most commonly overused agents were caffeine (50, 69.7%), acetaminophen (48, 66.7%), and aspirin (24, 33.3%).

**Table 1:**
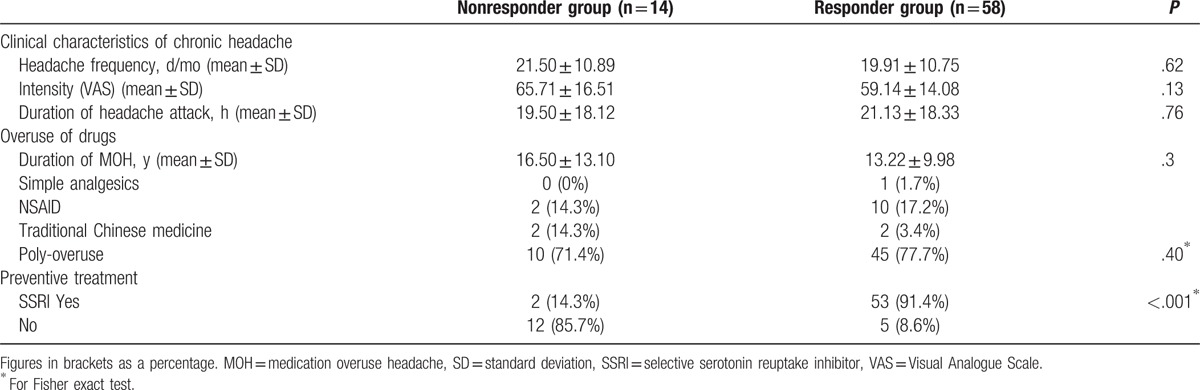
Clinical features of MOH patients.

All 72 patients’ medical records during withdraw therapy were reviewed to investigate preventive treatment. Most of the patients used SSRI as the preventive treatment during withdraw period. Few patient used tricyclic antidepressants, topiramate, and valproic acid as the preventive treatment.

### 3.1 Univariate analysis

#### 3.1.1 Demographic and sociological

We found no significant difference between responder group and nonresponder group (Table 2).

**Table 2:**
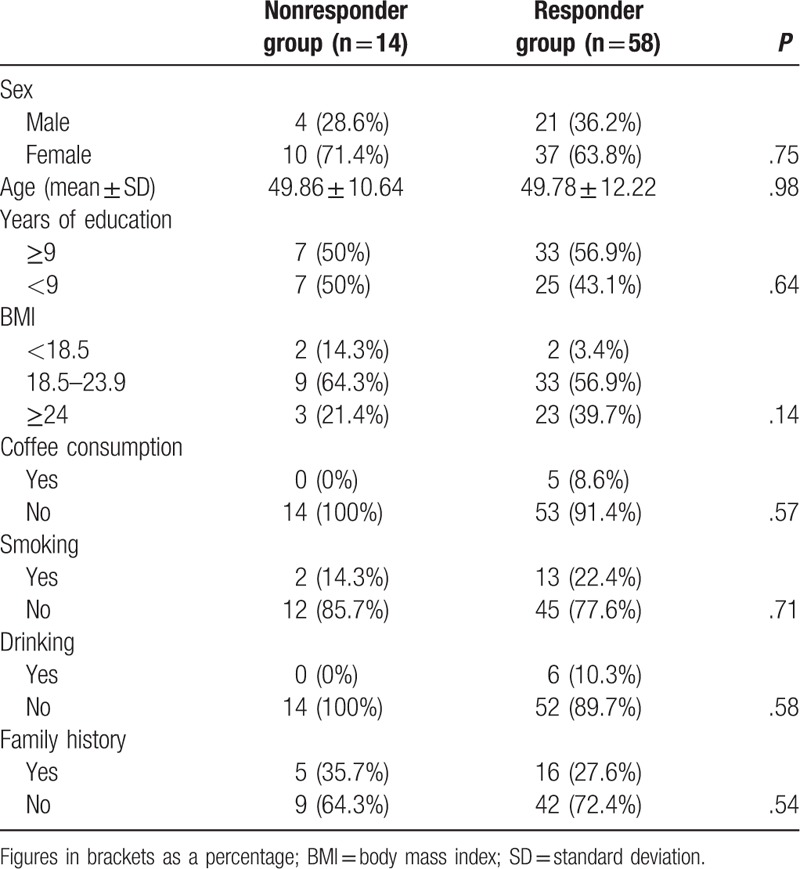
Baseline characteristics of patients that completed the study.

When analyzing the responder and nonresponder groups, we found no significant difference in chronic headache characteristics, overuse of drugs. However, when we compared the use of SSRIs, there was a significant difference between the 2 groups (Table 1).

### 3.2 Multivariate analysis

Univariate analysis showed that the use of SSRI was associated with treatment effect. So we perform the logistic regression analyses to test the association between the treatment effect and SSRI use. Logistic regression analyses suggested that SSRI use was an independent predictor of treatment effect (odds ratio OR: 0.016, 95% confidence interval [CI]: 0.003, 0.091, *P* < .001) (Table 3).

**Table 3:**
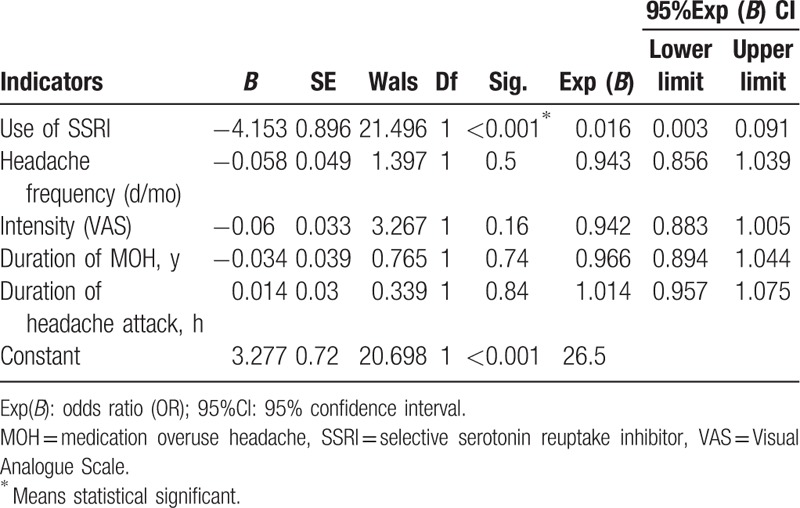
Multivariate unconditioned logistic regression analysis results.

Among 58 responders, 14 patients relapsed. We compared the demographic data between the non-relapser and relapser (Table 4). We found no significant difference between the 2 groups (Table 4). Cox regression analysis was used to test the association between the relapse and SSRI use. We found the use of SSRI was an important predictor of MOH outcome (hazard ratio [HR] = 0.255, 95%CI:0.09–0.724) (Table 5).

**Table 4:**
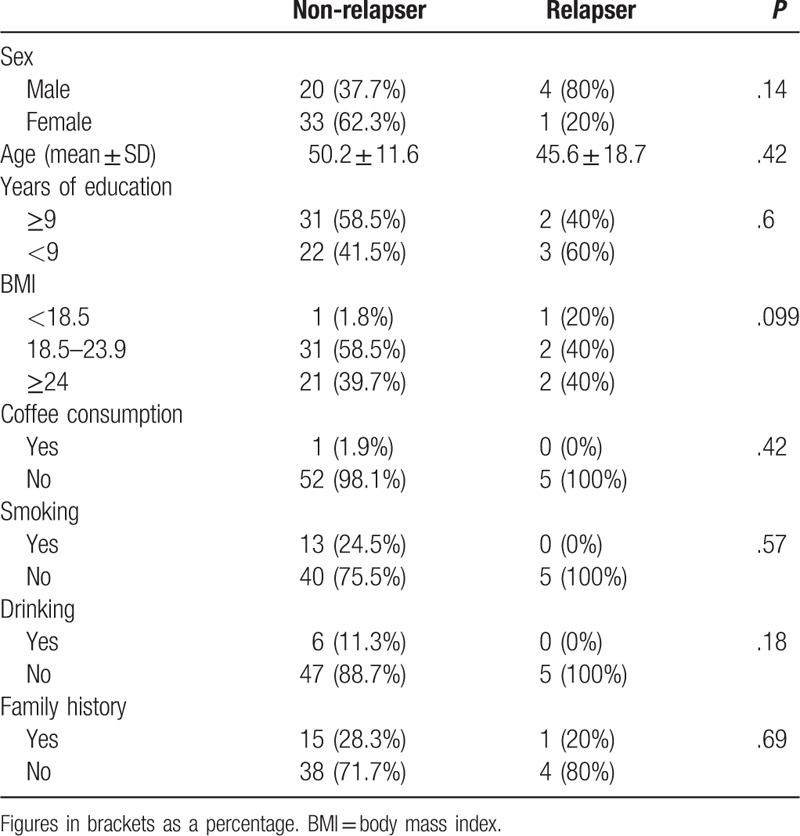
Baseline characteristics comparison between relapser and non-relapser.

**Table 5:**
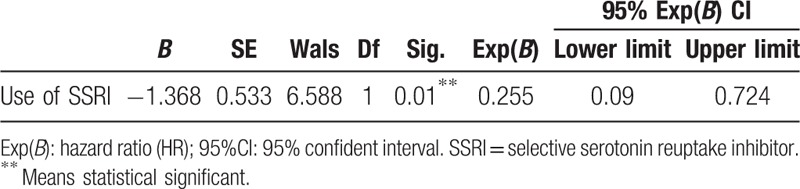
Cox regression analysis to test the association between SSRI use and relapse.

## 4 Discussion

Our study showed that the use of SSRIs in MOH withdrawal therapy reduces the risk of treatment failure in patients. In the treatment of depression and other related diseases, SSRI are widely used because its small side effects and better tolerance.^[21,22]^ However, previous studies suggested that SSRIs had no significant effect on treatment of a chronic migraine and tension-type headache.^[22]^ In the treatment of MOH, a case study showed that SSRI treatment had a significant effect on MOH including headache intensity decreased, quality of life improved.^[13]^ However, the number of patients in that study was small,^[13]^ and that study was an exploratory trial. In addition, many studies have shown that withdrawal therapy combined with early use of preventive treatment can significantly improve the quality of life of patients. MOH patients’ anxiety and depression symptoms are also significantly improved.^[23,24]^ Our study may provide the use of SSRIs as a new option for preventive treatment.

We found that the use of SSRIs during withdrawal therapy reduced the risk of relapse. In previous studies, the risk factors for MOH relapse including sex, compound analgesic drugs, nicotine, and alcohol abuse.^[16,25]^ And our study found that MOH long-term relapse have no significant correlation with demographic data including sex, education years, BMI, coffee consumption, drinking and family history of headache. No studies have yet shown that the use of SSRIs in withdrawal therapy can reduce the risk of relapse in patients with MOH. This is also our main finding of this study.

There are many limitations in this study: first, the study sample size is small, this study is only a single-center follow-up study, the patient's treatment effect need to be further investigate; Second, we didn’t assess MOH patients depression and anxiety score at the baseline. This is an outpatient study and patients’ compliance may lead to bias. Third, we did not record adverse effects when patient using SSRI during withdrawal treatment. Finally, the design of this study was retrospective study, and it cannot avoid other interference factors and lead to bias.

## 5 Author contributions

**Conceptualization:** J. Fang, Y. Zhang, L. He.

**Data curation:** J. Fang, Y. Zhang, N. Chen, J. Guo, L. He.

**Formal analysis:** J. Fang, Y. Zhang, N. Chen. J. Guo.

**Funding acquisition:** J. Fang.

**Investigation:** J. Fang, Y. Zhang, N. Chen, M. Zhou.

**Methodology:** J. Fang, Y. Zhang, N. Chen, J. Guo, M. Zhou.

**Project administration:** L. He.

**Resources:** M. Zhou, L. He.

**Software:** J. Guo, M. Zhou.

**Supervision:** L. He.

**Writing – original draft:** J. Fang.

**Writing – review & editing:** Y. Zhang, L. He.
